# Transcriptome profiling provides insights into molecular mechanism in Peanut semi-dwarf mutant

**DOI:** 10.1186/s12864-020-6614-0

**Published:** 2020-03-05

**Authors:** Fengdan Guo, Junjie Ma, Lei Hou, Suhua Shi, Jinbo Sun, Guanghui Li, Chuanzhi Zhao, Han Xia, Shuzhen Zhao, Xingjun Wang, Yanxiu Zhao

**Affiliations:** 1 College of Life Science, Shandong Normal University, Jinan, People’s Republic of China; 2 Biotechnology Research Center, Shandong Academy of Agricultural Sciences, Shandong Provincial Key Laboratory of Crop Genetic Improvement, Ecology and Physiology, Jinan, People’s Republic of China; 3 Life Science College of Shandong University, Jinan, 250100 People’s Republic of China

**Keywords:** Peanut (*Arachis hypogaea* L.), Main stem height, RNA-seq, Cell wall related genes, Hormones, Transcription factors

## Abstract

**Background:**

Plant height, mainly decided by main stem height, is the major agronomic trait and closely correlated to crop yield. A number of studies had been conducted on model plants and crops to understand the molecular and genetic basis of plant height. However, little is known on the molecular mechanisms of peanut main stem height.

**Results:**

In this study, a semi-dwarf peanut mutant was identified from ^60^Co γ-ray induced mutant population and designated as semi-dwarf mutant 2 (*sdm2*). The height of *sdm2* was only 59.3% of its wild line Fenghua 1 (FH1) at the mature stage. The *sdm2* has less internode number and short internode length to compare with FH1. Gene expression profiles of stem and leaf from both *sdm2* and FH1 were analyzed using high throughput RNA sequencing. The differentially expressed genes (DEGs) were involved in hormone biosynthesis and signaling pathways, cell wall synthetic and metabolic pathways. BR, GA and IAA biosynthesis and signal transduction pathways were significantly enriched. The expression of several genes in BR biosynthesis and signaling were found to be significantly down-regulated in *sdm2* as compared to FH1. Many transcription factors encoding genes were identified as DEGs.

**Conclusions:**

A large number of genes were found differentially expressed between *sdm2* and FH1. These results provide useful information for uncovering the molecular mechanism regulating peanut stem height. It could facilitate identification of causal genes for breeding peanut varieties with semi-dwarf phenotype.

## Background

Plant height, a decisive factor of plant architecture, is an important agronomic trait which contributes to crop yield. Plant height is highly associated with rain or wind caused lodging and the utilization of dwarf and semi-dwarf plants could be a way to reduce the risk. Elongation of main stem is the main limiting factor of plant height. The breeding of semi-dwarf rice and wheat varieties in 1960s gave rise to the first ‘green revolution’ and increased grain yields significantly [1, 2]. Peanut (*Arachis hypogaea* L.) is an important oil crop in the world. In China, most of cultivated peanut varieties are erect. To prevent overgrowth and lodging, growth inhibitory substances like paclobutrazol and uniconazole are usually applied 2~3 times during the growth season. Therefore, studying the underlying molecular mechanism controlling main stem height and developing semi-dwarf peanut variety are of great significance.

Stem elongation is determined by cell growth including cell division and cell expansion. The dynamic variation of plant cell wall is critical in these processes [3]. The cellulose synthase super-family containing cellulose synthases (CesA) and cellulose synthase-like genes (CSL) is very important in cell-wall biogenesis and remodeling. *CesA* or *CSL* mutation in plants resulted in a dwarf phenotype with short internode or hypocotyl and small leaves [4–8]. Cell wall extensibility controls the rate of plant cell growth, especially cell expansion [9]. Expansins are plant cell-wall loosening proteins which promote cell enlargement by influencing cell wall extensibility and are essential in many critical developmental processes [10]. Suppression of expansin genes resulted in small, curled leaves, reduced plant height and early flowering [11–14]. Besides expansins, xyloglucanendotransglucosylase/endohydrolases (XTHs) were also suggested to regulated cell wall loosening [3, 15]. XTHs catalyze the endo cleavage of xyloglucan polymers and subsequent restructuring, which allow cellulose microfibrils moving along with the cell expansion or elongation process [16–18]. EGases (endo-1,4-β-glucanases) have been proposed to modify hemicellulose network, and were involved in processes that require cell wall weakening, including cell elongation, organ abscission, and fruit softening [19].

A number of mutants with deficiencies in phytohormone biosynthesis and signal transduction displayed dwarf phenotype [20–24]. Extensive studies showed that most of the dwarf phenotype of plants were associated with gibberellins (GAs) and brassinosteroids (BRs), and a few were caused by auxin (IAA) or strigolactone (SL) [9]. Two “Green Revolution” genes, *sd1* and *Rht*, which encoded GA 20-oxidases and DELLA protein were cloned from rice and wheat, respectively [1, 2, 25, 26]. Later, several key genes controlling plant height have been identified. GID1 (GA-insensitive dwarf1) is the GA receptor, and its mutant *gid1* in rice showed dwarf phenotype [27]. GA3-oxidases (GA3ox) catalyze the synthesis of bioactive GAs and GA2-oxidases (GA2ox) convert excess GAs to inactive forms. *GA3ox* deletion and *GA2ox* overexpression both reduced the plant height [28, 29].

Mutants of BR signaling related genes, for examples, the *deetiolated* (*det*) [30], *diminuto* (*dim*) [31], *constitutive photomorphogenesis and dwarfism* (*cpd*) [23] and *dwarf4* (*dwf4*) [32] are all dwarf. *DIM* was also referred to as *DWF1* or *CBB1*, could convert 24-methylenecholesterol to campesterol [31, 33]. *DET2* was shown to encode a putative steroid 5*α*-reductase [34, 35]. *CPD* and *DWF4* were members of cytochrome P450 family, encoding a putative C-3 oxidase and 22*a*-hydroxylase, respectively [32, 36]. BRI1 (Brassinosteroid Insensitive 1) is the membrane-localized BR receptor [37–39]. The *bri1* mutant displayed severely dwarf phenotype and reduced apical dominance [40]. BAK1 (BRI1-Associated Receptor Kinase 1) is co-receptor of BRI1 and can trigger sequential transphosphorylation of BRI1/BAK1 receptor kinase complex [41–43]. The null allele of BAK1 displayed a semi-dwarf phenotype and reduced sensitivity to BRs. BIN2 plays a negative regulatory role in BR signaling and its gain-of-function mutant showed the phenotype similar to BR-deficient and BR-response mutants [44]. BES1 and BZR1 are two bHLH transcription factors, and they can directly bind to target gene promoters together with other transcription regulators to activate BR target gene expression [24, 45–48].

Auxin polar transport is critical in regulating the whole progress of plant growth and development [49]. AUX1/LAX family, PIN family and ABCB subfamily are three classes of polar auxin transporters. *MDR* (Multidrug Resistance) genes encode P-glycoproteins and belong to subfamily B of ATP-binding cassette (ABCB). The *atmdr1* mutants were defective in basipetal auxin transport and shorter than wild-type plants [50]. SAUR (Small Auxin up RNA) genes, the largest family of auxin-responsive genes, functioned as positive effectors of cell expansion and were up-regulated in auxin-mediated cell elongation [51–53].

Many transcription factors are important regulators of plant height. *SHORT INTERNODES* (*SHI*) gene is a negative regulator of GA response and its overexpression mutant *shi* showed a dwarf phenotype similar to mutants defective in GA biosynthesis or response [54]. *WRKY46/54/70* could be activated by BRs and were cofactors of BES1 to modulate BR target genes [55]. PACLOBUTRAZOL RESISTANT (PRE) is a positive regulator of cell elongation which can be activated by BRs, GAs and repressed by light [56, 57]. PRE together with IBH1, HBI1 and ACE constitute a triantagonistic cascade module which integrate hormonal and environmental signals to regulate cell elongation [58–60].

In peanut, genetic basis underlying plant height remains unclear. Some studies to identify QTLs for plant height have been conducted in peanut. Huang et al. (2016) constructed a high-density genetic linkage map containing 1219 mapped loci using a recombinant inbred line (RIL) population and identified 18 QTLs for plant height [61]. Li et al. (2017) detected 11 QTLs for main stem height using a RIL population [62]. Using two RIL populations, Lv et al. (2018) detected 11 QTLs for plant height. Among which, two QTLs from these two populations were co-localized in a physical interval of 3.4 Mb on A09 [63]. However, no gene closely related to plant height has been cloned in peanut until now.

In the previous research, we built a mutant library through ^60^Co radiation. Among which, two mutants displayed semi-dwarf phenotype. In this study, genome-wide gene expression profiling of stem and leaf from *sdm2* was conducted to understand the underlying regulation network of main stem height in peanut. The expression of several genes involved in biosynthesis and signal transduction of BRs and GAs were drastically altered in *sdm2*. Accordingly, the downstream genes related to cell wall biogenesis and remodeling were also changed at the transcriptional level.

## Results

### Phenotype characteristics of peanut dwarf mutant

**Fig. 1:**
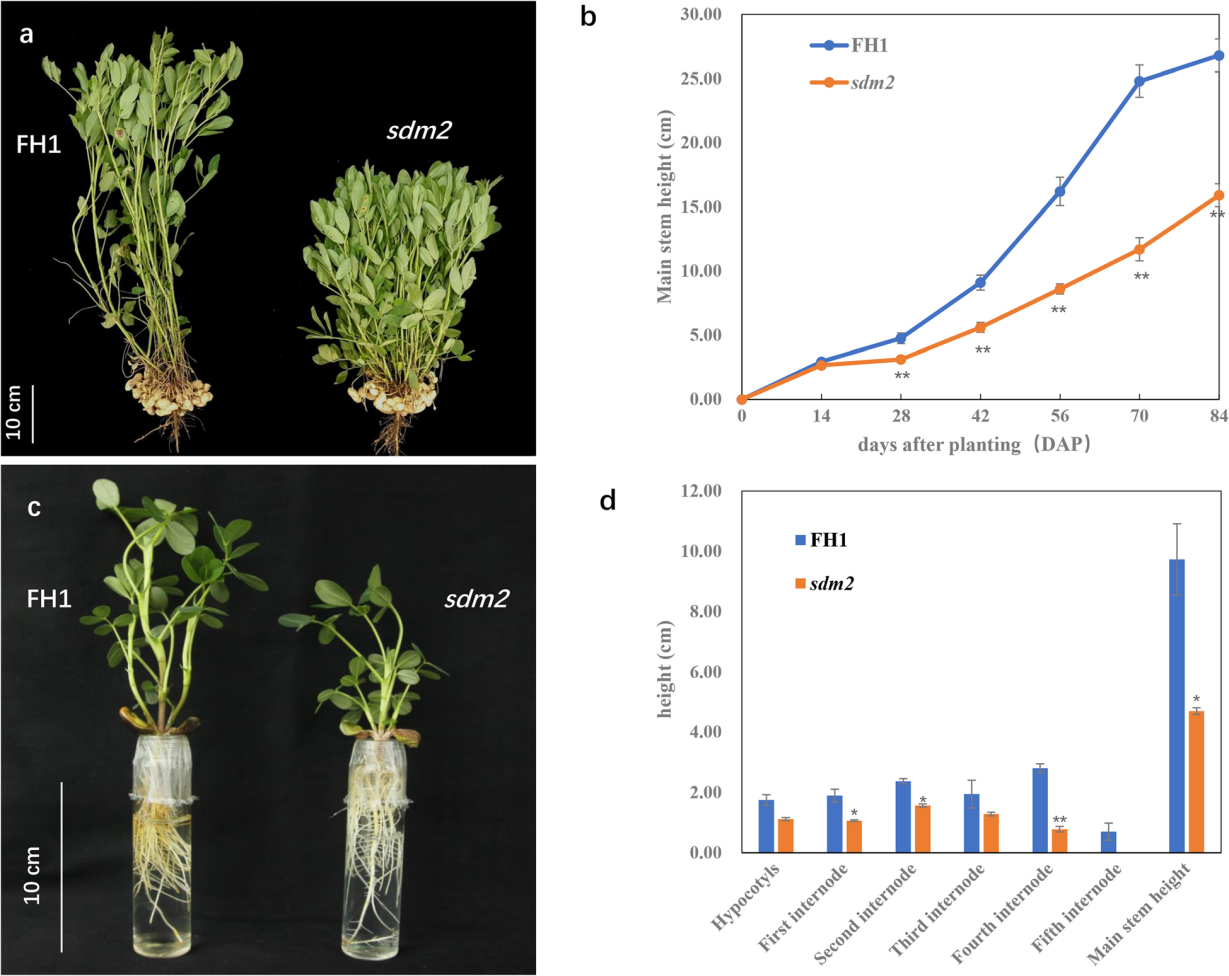
Phenotypic characterization of *sdm2* mutant and FH1. **a** Plants of *sdm2* and FH1 at harvest time grown in the field; **b** Main stem height of *sdm2* and FH1 at different growth stages grown in the field; **c** *sdm2* and FH1 grown in Hogland solution for 14 DAP; **d** Main stem height measurement of *sdm2* and FH1 grown in Hogland solution for 14 DAP. Three biological replicates were used for statistical analysis (t-test; *P < 0.05, **P < 0.01). Values in b, d represent means ± SE (*n* = 3)

The main stem height of *sdm2* is significantly shorter than that of FH1 (*P* < 0.01) (Fig. 1a). We measured the main stem height of mutant and FH1 grown in the experimental farm every 2 weeks during the vegetative growing stage. Results showed that during the first 2 weeks, main stem height between *sdm2* and FH1 had no obviously difference. At 28 DAP (days after planting), main stem height of *sdm2* was significantly shorter than that of FH1 (*P* < 0.01). In the following developmental stages, the height difference between *sdm2* and FH1 became more and more obvious. At 84 DAP, the *sdm2* was 15.9 cm in height, only about 60% of FH1 (26.8 cm) (Fig. 1b). The leaf of *sdm2* was smaller, thicker and dark green in color, and showed delayed senescence compare to that of FH1. At harvest time, the number of internodes of *sdm2* was 3~4 less than FH1. The number of branches (*P* < 0.05) and pods (*P* > 0.05) per plant was obviously more, while the 100-pod weight and 100-kernel weight were significantly decreased in *sdm2* (*P* < 0.01).

To further accurately measure the change of mutant main stem height, we conducted hydroponic culture of *sdm2* and FH1 in Hogland solution. The length of each internode and hypocotyl of 14 DAP seedlings were measured. We found that it was different from the results in experimental farm. For 14 DAP seedlings, the *sdm2* height was already significantly shorter than that of FH1 (*P* < 0.05) (Fig. 1c, d). There were total five internodes in FH1 and four in *sdm2* seedlings. The hypocotyl length of *sdm2* was slightly shorter than FH1 and there was no significant difference (*P* > 0.05) for the 14 DAP seedlings. The length of most internodes of *sdm2* was significantly shorter than FH1 (Fig. 1d). These results indicated that the height difference between *sdm2* and FH1 was caused by both reduced total number of internode and length of each internode.

### Sequencing data analysis

To clarify global gene expression changes in *sdm2*, 12 cDNA libraries were constructed with stem and leaf from mutant and FH1 seedlings. The libraries were sequenced using BGISEQ-500 platform. A total of 0.26 billion raw reads were generated, and the average output of each sample was 21.7 million. After removing adaptor sequences, low-quality and N-containing reads, an average of 21.58 million clean reads were obtained from each library with the clean read ratio of 99.47% (Table 1). Approximately 19.27 million and 16.00 million clean reads in each library matched the reference genome and gene set perfectly with the average mapping ratio of 89.31 and 74.18%, respectively. To investigate gene expression correlation among samples, the Pearson correlation coefficient of all gene expression levels between each two samples were calculated (Additional file 1: Fig. S1). It showed a higher correlation of similar tissue between *sdm2* and FH1. The principal component analysis (PCA) could clearly divide all samples into two clusters according to two kinds of tissues (Additional file 2: Fig. S2).

**Table 1:** Summary of read numbers from stem and leaf in *sdm2* and FH1

Sample name	Reads in stem						Reads in leaf					
	FH1_S_1	FH1_S_2	FH1_S_3	*sdm2*_S_1	*sdm2*_S_2	*sdm2*_S_3	FH1_L_1	FH1_L_2	FH1_L_3	*sdm2*_L_1	*sdm2*_L_2	*sdm2*_L_3
Total raw read(M)	21.64	21.66	21.67	21.65	21.84	21.67	21.86	21.61	21.74	21.65	21.64	21.71
Total clean read(M)	21.51	21.54	21.56	21.53	21.74	21.52	21.78	21.50	21.65	21.47	21.53	21.60
Clean read ratio(%)	99.41	99.46	99.48	99.48	99.56	99.30	99.60	99.51	99.60	99.19	99.50	99.49
Clean read q20(%)	98.25	98.22	98.16	98.20	98.06	98.07	98.27	98.15	98.21	98.31	98.11	98.24
Clean read q30(%)	90.52	90.37	90.10	90.36	89.52	89.81	90.28	90.06	90.11	90.76	89.85	90.29
Total mapping genome reads (M)	20.34	20.53	20.72	20.56	20.82	20.15	17.95	17.05	19.40	19.03	16.41	18.30
Total mapping genome ratio(%)	94.56	95.33	96.09	95.49	95.79	93.63	82.42	79.30	89.60	88.65	76.20	84.70
Total mapping gene reads (M)	16.93	16.75	17.45	17.17	17.32	16.46	14.63	14.24	15.77	16.41	13.93	15.02
Total mapping gene ratio (%)	78.69	77.77	80.95	79.73	79.65	76.51	67.17	66.24	72.86	76.45	64.68	69.56

Note: S indicates stem, and L indicates leaf. Each sample has three replicates

### DEGs between *sdm2* and FH1

**Fig. 2:**
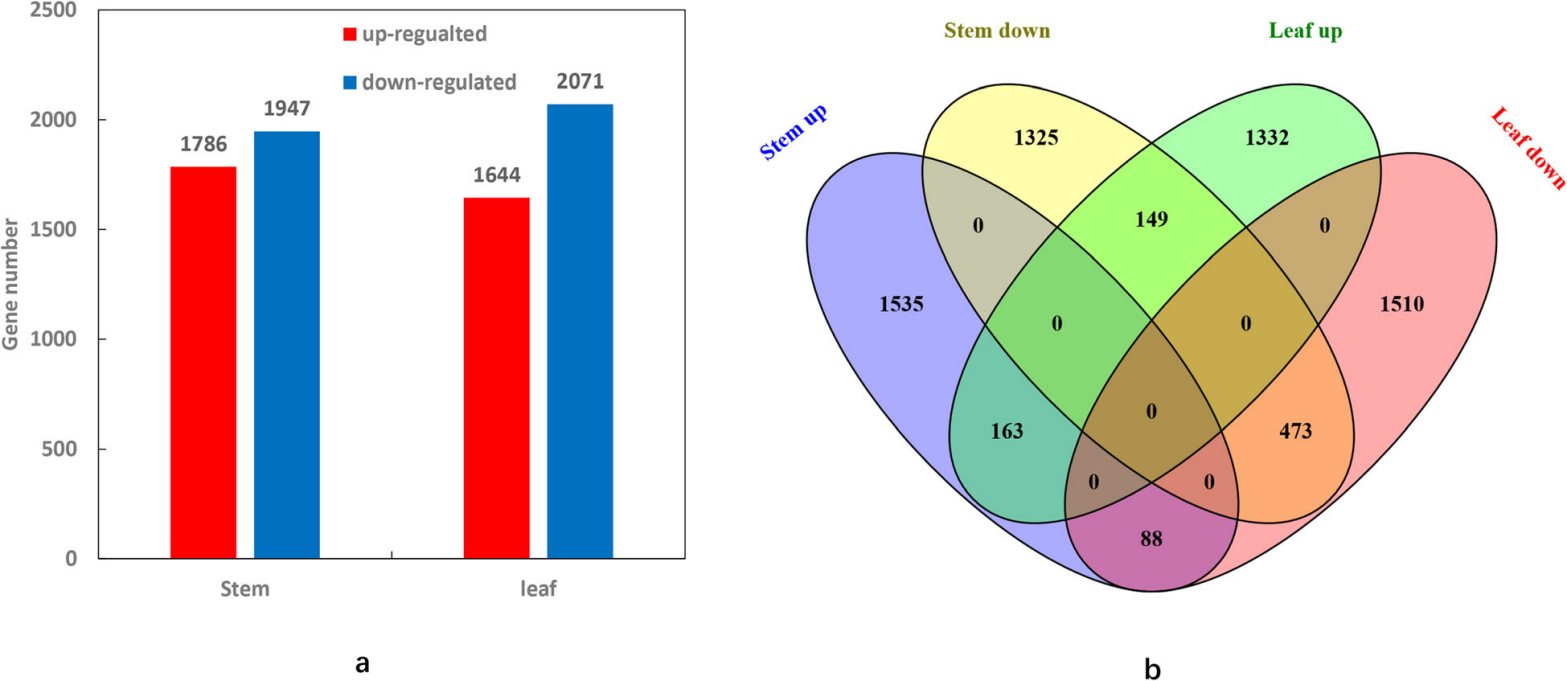
Differentially expressed genes in *sdm2* and FH1. **a** Numbers of differentially expressed genes in *sdm2* and FH1; **b** Venn diagram showed the common and specific differentially expressed genes in stem and leaf

From all the samples, a total of 65,435 genes were identified. The gene expression of stem and leaf between *sdm2* and FH1 was analyzed, and 3733 and 3715 differentially expressed genes (DEGs) were identified, respectively (Additional files 3 and 4: Table S1 and S2). In *sdm2* stem, 1786 genes were up-regulated and 1947 genes were down-regulated to compare with FH1 (Fig. 2a and Additional file 3: Table S1). In *sdm2* leaf, the up- and down-regulated genes were 1644 and 2071, respectively (Fig. 2a and Additional file 4: Table S2). The majority of DEGs had different expression pattern in stem and leaf. Among 3733 DEGs in stem, only 873 were also differentially expressed in leaf. There were 473 down-regulated and 163 up-regulated DEGs with the same change trend in stem and leaf. It is worth noting that the number of common down-regulated DEGs was obviously higher than that of up-regulated ones. Interestingly, a large number of DEGs showed opposite expression pattern in stem and leaf. For example, there were 149 genes down-regulated in mutant stem while up-regulated in leaf and 88 genes up-regulated in mutant stem but down-regulated in leaf (Fig. 2b).

### Functional analysis of DEGs

**Fig. 3:**
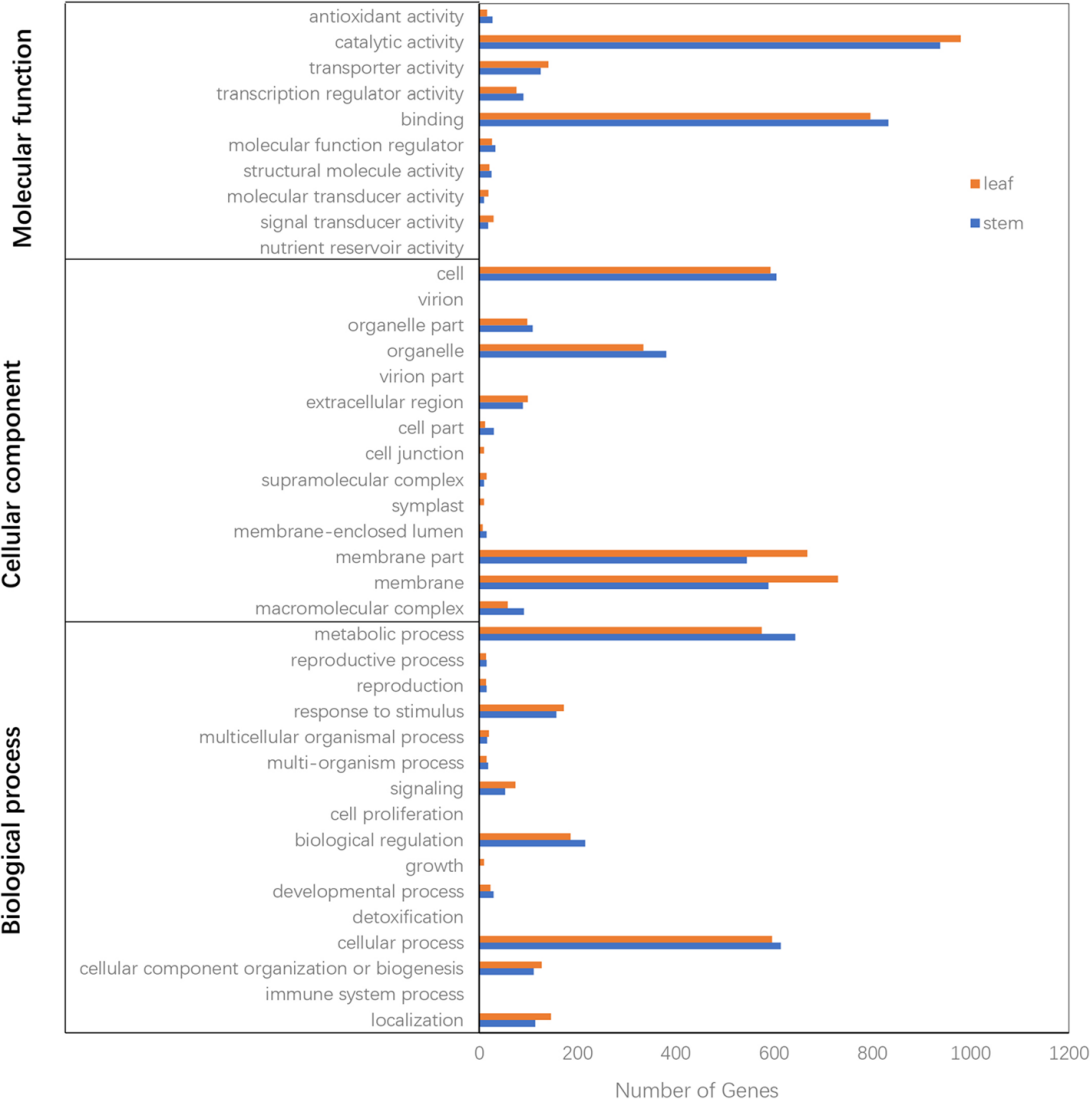
GO classification of DEGs in stem and leaf. The X-axis represents the number of genes annotated into the GO terms, and the Y-axis represents the functional classification

GO classification showed that 1724 DEGs from stem and 1716 DEGs from leaf were classified into three categories: biological process, cellular component, and molecular function (Fig. 3 and Additional file 5: Table S3). In molecular function category, catalytic activity and binding were the most abundant terms. For cellular component category, cell, membrane, membrane part and organelle were the main terms. The top two terms of biological process were metabolic process and cellular process. The top 20 terms in stem and leaf were shown in Additional file 6: Fig. S3 and Additional file 7: Table S4.

**Fig. 4:**
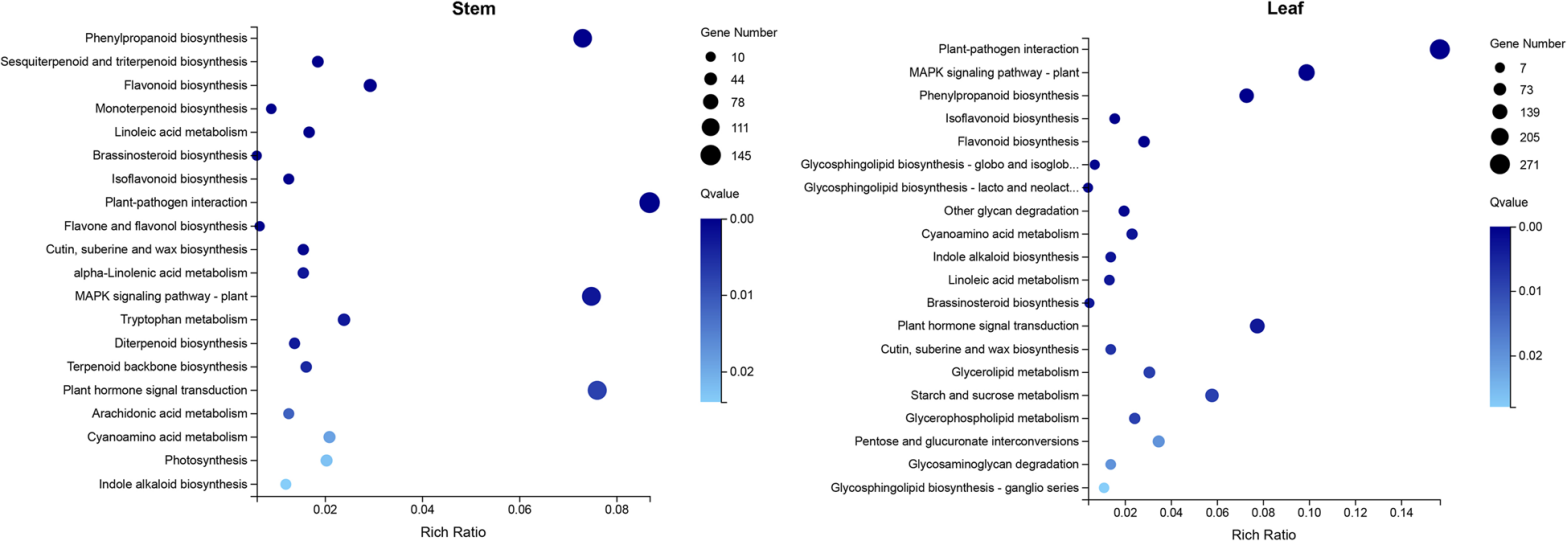
Bubble diagram of top 20 enriched KEGG pathways of DEGs in stem and leaf. X axis represents the Rich Ratio, which meaning the ratio of selected gene number annotated to a particular item to the total number of genes in this item in one species. The calculating formula is Rich Ratio = Term Candidate Gene Num/Term Gene Num. Y axis represents KEGG Pathway. The size of the bubbles indicates the number of genes annotated to a KEGG Pathway. And the color represents Q-value of enrichment. The deeper the color, the smaller the Q-value

KEGG pathway analysis was also carried out. According to the enrichment results, the top 20 pathways in stem and leaf were shown in Fig. 4 and Additional file 8: Table S5. In stem, many pathways involved in hormone biosynthesis and signal transduction including terpenoid backbone biosynthesis, monoterpenoid biosynthesis, diterpenoid biosynthesis, sesquiterpenoid and triterpenoid biosynthesis, zeatin biosynthesis, and brassinosteroid biosynthesis, indole alkaloid biosynthesis as well as plant hormone signal transduction were all enriched in the mutant (Fig. 4 and Additional file 8: Table S5). In addition, MAPK signaling pathway was also enriched. These results suggested that plant hormone play major roles in regulation of the mutant phenotype. There were other pathways including photosynthesis, phenylpropanoid biosynthesis, flavonoid biosynthesis, isoflavonoid biosynthesis, and flavone and flavonol biosynthesis pathways were found to be enriched.

In leaf, brassinosteroid biosynthesis, indole alkaloid biosynthesis, plant hormone signal transduction and MAPK signaling pathways were enriched in accordance with those in stem (Fig. 4 and Additional file 8: Table S5). Phenylpropanoid biosynthesis, flavonoid biosynthesis, isoflavonoid biosynthesis and plant-pathogen interaction were also in the top 20 enriched pathways. Different from that in stem, some glucide and glucolipid metabolic pathways were enriched in leaf.

### Cell wall related genes

**Fig. 5:**
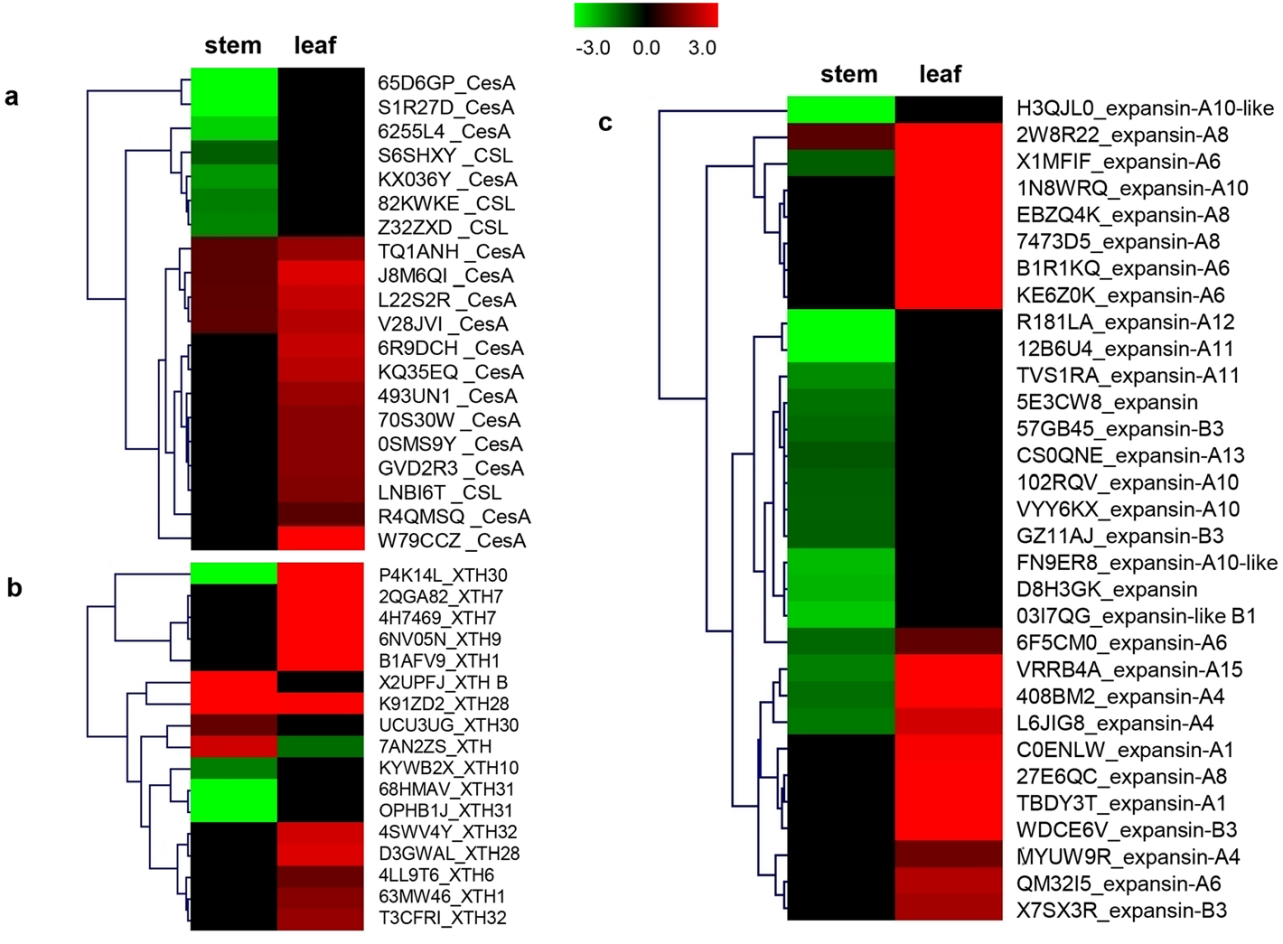
The expression pattern of cell wall related genes in stem and leaf of *sdm2*. Heatmaps represent the differential expression of *CesA* and *CSL* **a**, *XTHs* **b**, *expansions* **c** in stem and leaf between *sdm2* and FH1. Scale bar is located at upside with log2 ratio value varying from green to red

RNA-seq results showed that several *CesA* genes and all *CSL* genes were down-regulated in stem. The expression of most expansin genes was down-regulated in *sdm2* stem compared with those in FH1 (Fig. 5 and Additional file 3: Table S1). Interestingly, the expression of *CesA*, *CSL* and *expansin* genes was up-regulated in leaf (Fig. 5 and Additional file 4: Table S2). These results were coincided with the phenotype of *sdm2* leaf, dark green in color, thicker, smaller than FH1 leaf.

Some *XTHs* were significantly up-regulated and some were down-regulated in *sdm2* stem (Fig. 5 and Additional file 3: Table S1). In leaf, the expressions of all *XTHs* were up-regulated, and some *XTHs* were 16-fold higher than those in FH1 (Fig. 5 and Additional file 4: Table S2). Some genes encoding endo-1,4-β-glucanases (EGases) in stem and leaf were down-regulated.

### Expression changes of hormone biosynthesis and signaling genes

DWF4 was designated as CYP90B1 and proposed to catalyze steroid 22*α-*hydroxylation in BR biosynthesis [64]. A gene annotated as cytochrome P450 90B1 showed 73% identity with Arabidopsis *DWF4* gene, which play key roles in BR biosynthesis. In stem, the expression of this gene was detected in FH1, while not detected in *sdm2.* In leaf, the expression of this gene in *sdm2* was also lower than that in FH1. Previous studies confirmed that cytochrome P450 monooxygenase CYP90A1 (CPD) acted as BR C-3 oxidase [36]. The expression level of *CYP90A1* (*CPD*) in *sdm2* stem decreased by 1.2-fold than that in FH1. While in leaf, its expression was slightly higher in *sdm2* than in FH1. The cytochrome P450 family members, CYP90C1/D1, CYP85 (*Dwarf*) and CYP71A1, mediate rate-limiting reactions in BR biosynthesis [65–67]. Our results showed that the expression of *CYP90C1/D1* gene decreased by 2.64-fold and 1.41-fold in stem and leaf respectively in *sdm2*. The *Dwarf* genes which encoding cytochrome P450 85A-like in stem and cytochrome P450 85A in leaf were all up-regulated. CYP71A1 which catalyze the generation of deoxocastasterone (6-deoxoCS) and castasterone (CS) was down-regulated both in stem and leaf in *sdm2*. A cytochrome P450 monooxygenase (CYP734A1) which degrades BRs was up-regulated in *sdm2* leaf (Table 2, Additional files 3 and 4: Table S1 and S2).

**Table 2:** Differential expression of hormonal biosynthesis and signaling genes in stem and leaf between *sdm2* and FH1

Gene ID	Nr Annotation	Gene name	Relative expression level (*sdm2*/FH1)				Expression trend (stem/leaf)
			log_2_(stem)	Q-value (stem)	log_2_(leaf)	Q-value (leaf)	
Arahy.MA1Z2D	cytochrome P450 90B1	DWF4	−9.35	2.43E-59	−3.99	1.29E-06	down/down
Arahy.66GL51	cytochrome P450 90A1 isoform X1	CPD	−1.31	9.68E-13	–	–	down/−
Arahy.7AH9CA	3-epi-6-deoxocathasterone 23-monooxygenase	CYP90C1/D1	−2.40	2.29E-13	−1.59	2.47E-12	down/down
Arahy.31R89H	cytochrome P450 85A-like	Dwarf/BR6ox	1.99	1.08E-07	–	–	up/−
Arahy.JZF5VE	cytochrome P450 85A-like	Dwarf/BR6ox	2.50	9.71E-13	–	–	up/−
Arahy.27TAQT	cytochrome P450 85A	Dwarf/BR6ox	–	–	2.16	4.98E-31	−/up
Arahy.2F8F1S	cytochrome P450 85A	Dwarf/BR6ox	–	–	1.34	2.56E-35	−/up
Arahy.TI8LFW	cytochrome P450 71A1	CYP71A	−1.81	0.00E+ 00	−2.06	2.09E-53	down/down
Arahy.ZW245F	cytochrome P450 71A1-like	CYP71A	−1.63	3.04E-32	–	–	down/−
Arahy.U2CXJI	cytochrome P450 734A1	BAS1	–	–	1.80	0.00E+ 00	−/up
Arahy.GJFA3Z	probable leucine-rich repeat receptor-like protein kinase At5g49770 isoform X1	BRI1	−2.38	3.16E-51	–	–	down/−
Arahy.56X5WL	probable leucine-rich repeat receptor-like protein kinase At5g49770 isoform X1	BRI1	−1.32	1.91E-44	–	–	down/−
Arahy.BMJ2CS	probable leucine-rich repeat receptor-like protein kinase At1g35710	BRI1	–	–	−2.59	6.71E-07	−/down
Arahy.69MPHZ	probable leucine-rich repeat receptor-like protein kinase At5g49770	BRI1	–	–	−2.49	7.76E-19	−/down
Arahy.DH99G0	BRASSINOSTEROID INSENSITIVE 1-associated receptor kinase 1-like isoform X1	BAK1	−1.99	4.09E-07	−2.08	2.39E-74	down/down
Arahy.SPI7HB	BES1/BZR1 homolog protein 2	BES1/BZR1	−8.42	0.00E+ 00	−7.30	0.00E+ 00	down/down
Arahy.RCTC3C	BES1/BZR1 homolog protein 2	BES1/BZR1	−1.92	7.59E-08	−1.20	1.08E-04	down/down
Arahy.ND2ZET	BES1/BZR1 homolog protein 2	BES1/BZR1	−1.94	4.11E-05	–	–	down/−
Arahy.22IVZ0	gibberellin 20 oxidase 1-D	GA20ox	5.77	3.55E-98	–	–	up/−
Arahy.B5269G	gibberellin 20 oxidase 1-like	GA20ox	1.64	8.35E-05	–	–	up/−
Arahy.BLJ7SD	gibberellin 20 oxidase 2	GA20ox	5.60	4.62E-85	–	–	up/−
Arahy.YI9B6B	probable 2-oxoglutarate-dependent dioxygenase At5g05600 isoform X1	2-oxoglutarate-dependent dioxygenase	1.39	2.07E-06	1.23	1.02E-04	up/up
Arahy.XPP88F	2-oxoglutarate-dependent dioxygenase AOP3	2-oxoglutarate-dependent dioxygenase	1.08	9.86E-04	−1.90	4.14E-23	up/down
Arahy.MT91AM	2-oxoglutarate-dependent dioxygenase AOP3	2-oxoglutarate-dependent dioxygenase	1.74	4.26E-07	–	–	up/−
Arahy.K8MVYG	gibberellin 3-beta-dioxygenase 1	GA3ox	−1.41	1.18E-49	–	–	down/−
Arahy.QH4I6C	gibberellin 2-beta-dioxygenase 1 isoform X1	GA2ox	−1.87	2.50E-04	−1.82	1.12E-11	down/down
Arahy.VR90R2	gibberellin 2-beta-dioxygenase 8	GA2ox	2.74	1.25E-17	–	–	up/−
Arahy.EPT56A	gibberellin-regulated protein 11	GRP11	−1.84	1.80E-09	–	–	down/−
Arahy.F1TUFL	gibberellin-regulated protein 4	GRP4	−1.63	0.00E+ 00	–	–	down/−
Arahy.FJ610L	gibberellin-regulated protein 4	GRP4	–	–	4.31	1.92E-70	−/up
Arahy.X9WA5F	PREDICTED: gibberellin-regulated protein 4	GRP4	−1.20	8.62E-115	3.90	3.18E-37	down/up
Arahy.VJ6NUA	gibberellin-regulated protein 6 isoform X2	GRP6	−1.44	1.83E-145	3.75	5.91E-89	down/up
Arahy.UA0Z8F	auxin transporter-like protein 1	AUX1/LAX family	6.46	2.83E-11	4.70	1.25E-15	up/up
Arahy.JN7ZD3	auxin transporter-like protein 1	AUX1/LAX family	3.59	1.64E-04	–	–	up/−
Arahy.LRNE2N	auxin transporter-like protein 3	AUX1/LAX family	–	–	2.06	4.38E-72	−/up
Arahy.953E9B	auxin efflux carrier component 5 isoform X1	PIN family	–	–	−3.06	3.04E-06	−/down
Arahy.K2GLG1	auxin efflux carrier component 5 isoform X1	PIN family	–	–	−1.36	2.22E-09	−/down
Arahy.BB2RVD	ABC transporter B family member 19-like	ABCB subfamily	3.17	1.24E-04	1.27	3.06E-04	up/up
Arahy.RLV4PD	ABC transporter B family member 1	ABCB subfamily	–	–	3.48	2.67E-09	−/up
Arahy.M63M99	ABC transporter B family member 11	ABCB subfamily	–	–	−1.27	2.65E-33	−/down
Arahy.YCYQ9Q	uncharacterized protein LOC107461514	ABCB subfamily	−5.97	7.24E-43	−7.49	2.35E-72	down/down
Arahy.M4RITN	uncharacterized protein LOC107461514	ABCB subfamily	−5.13	2.19E-18	−3.56	5.47E-04	down/down
Arahy.8QDS1I	auxin-induced protein AUX22-like	Aux/IAA protein	−2.12	5.87E-54	1.18	2.98E-30	down/up
Arahy.KRC5M1	auxin-induced protein AUX22	Aux/IAA protein	−1.52	3.07E-35	1.34	3.65E-35	down/up
Arahy.2A4XC9	LOW QUALITY PROTEIN: auxin-responsive protein IAA29	Aux/IAA protein	1.69	9.56E-15	1.79	1.13E-09	up/up
Arahy.S4Y3WA	auxin response factor 5 isoform X2	ARF	2.95	2.80E-24	2.97	1.67E-04	up/up
Arahy.1B1I67	putative auxin response factor 23	ARF	2.15	4.14E-10	–	–	up/−
Arahy.X5Q10C	auxin response factor 18-like isoform X2	ARF	–	–	5.44	5.42E-06	−/up
Arahy.MJW2EP	auxin-induced protein 15A-like	SAUR family	1.86	2.74E-04	6.59	5.68E-12	up/up
Arahy.E8WPL7	auxin-induced protein 6B-like	SAUR family	1.26	4.39E-05	2.93	2.63E-08	up/up
Arahy.W3E9BL	auxin-responsive protein SAUR50-like	SAUR family	−1.11	3.81E-13	3.98	1.86E-07	down/up
Arahy.NWIK25	indole-3-acetic acid-induced protein ARG7-like	SAUR family	–	–	4.77	2.09E-07	−/up
Arahy.G43XWA	protein SHORT INTERNODES-like	SHI	−6.32	1.91E-10	−6.63	3.98E-12	down/down
Arahy.F7N9UG	protein SHI RELATED SEQUENCE 3-like	SRS	5.43	6.62E-06	–	–	up/−
Arahy.R0GFR4	protein SHI RELATED SEQUENCE 3-like	SRS	–	–	−6.63	3.98E-12	−/down
Arahy.EXJ5K8	probable WRKY transcription factor 30	WRKY30	−1.20	7.10E-23	−3.09	1.42E-76	down/down
Arahy.TC7Y0P	probable WRKY transcription factor 46	WRKY46	−1.39	5.50E-62	−1.96	1.75E-96	down/down
Arahy.I9PJJN	probable WRKY transcription factor 70	WRKY70	−2.64	4.37E-05	−1.65	1.84E-26	down/down
Arahy.2031JR	transcription factor PRE6	PRE6	−2.41	5.06E-28	2.91	6.65E-10	down/up
Arahy.577H6Y	transcription factor PRE6	PRE6	−1.93	2.80E-07	6.24	4.25E-36	down/up
Arahy.HR6FN8	transcription factor PRE6	PRE6	−2.67	7.25E-71			down/−
Arahy.YX8FPP	transcription factor PRE6-like	PRE6	−2.33	5.39E-05			down/−
Arahy.WVIE7L	transcription factor PRE6	PRE6	–	–	7.87	2.15E-50	−/up

In both stem and leaf, the expression levels of *BRI1* and *BAK1* were down-regulated in *sdm2*. Noticeably, the expression levels of *BES1*/*BZR1* gene were extremely low in stem and leaf in *sdm2*, 170-fold and 79-fold lower than in FH1, respectively. The expression levels of *IWS1* in stem were up-regulated in *sdm2*, while in leaf, it was not differentially expressed. The expression of cyclin-D3–3 encoding gene was down-regulated in *sdm2* stem, which was coincident with the expression trends of BR-related genes. While the expression levels of *cyclin-D1* and *cyclin-D5* increased in *sdm2* leaf (Table 2, Additional files 3 and 4: Table S1 and S2).

Several GA biosynthesis and signal transduction genes were differentially expressed between *sdm2* and FH1. In *sdm2*, most of GA20ox and 2-oxoglutarate-dependent dioxygenase encoding genes, which promote GA biosynthesis, were up-regulated in stem, while most 2-oxoglutarate-dependent dioxygenase decreased in leaf. While two genes encoding GA3ox were down-regulated in stem and its differential expression was not detected in leaf. GA2ox convert excess GAs to inactive forms through 2β-hydroxylation [29]. In mutant stem and leaf, genes encoding GA2ox1 and GA2ox2 were decreased in expression levels while the expression of three genes encoding GA2ox8 were up-regulated in stem (Table 2, Additional files 3 and 4: Table S1 and S2).

Two *GID1* genes in stem showed opposite change trend and one *GID1* gene was down-regulated in leaf. *SCL3* (Scarecrow-like 3) seemed to attenuate DELLA protein and acted as a positive regulator in GA pathway [68]. In mutant stem, *SCL3* gene was up-regulated while two *SCL14* and one *SCL21* genes were down-regulated. Interestingly, all genes encoding gibberellin-regulated protein (GRP) were reduced in *sdm2* stem while up-regulated in leaf, which were consistent with the change trends of cell wall related genes including *CesA*, *CSL, expansin* and *XTH* genes (Table 2, Additional files 3 and 4: Table S1 and S2).

Four *AUX1* genes in stem and two in leaf were all significantly up-regulated, while the expressional levels of two genes encoding auxin efflux carrier (PIN) in leaf decreased. In addition, the expression change trends of *MDR/ABCB* genes in stem and leaf of *sdm2* were irregular, most of which were down-regulated and some were up-regulated. In *sdm2* stem, two genes encoding AUX22 were down-regulated and almost all ARF members were up-regulated, while in leaf, the expressions of all *Aux/IAA* and *ARF* genes were increased. In addition, most *SAUR* genes in stem and almost all *SAUR* genes in leaf were up-regulated in *sdm2* (Table 2, Additional files 3 and 4: Table S1 and S2).

### Transcription factors involved in plant growth and development

Our results showed that several transcription factor genes involved in hormone signaling pathways and their downstream changed significantly. Gene encoding SHORT INTERNODES-like protein was down-regulated both in stem and leaf of *sdm2*. Whereas, *SHI-RELATED SEQUENCE 3-like* (*SRS*) gene increased in stem and decreased in leaf. All *WRKY30/46/70* genes were down-regulated in *sdm2* stem and leaf. The expression levels of four *PRE6* genes decreased in stem and three *PRE6* genes were up-regulated in leaf (Table 2, Additional files 3 and 4: Table S1 and S2).

### Verification of DEGs using qRT-PCR

**Fig. 6:**
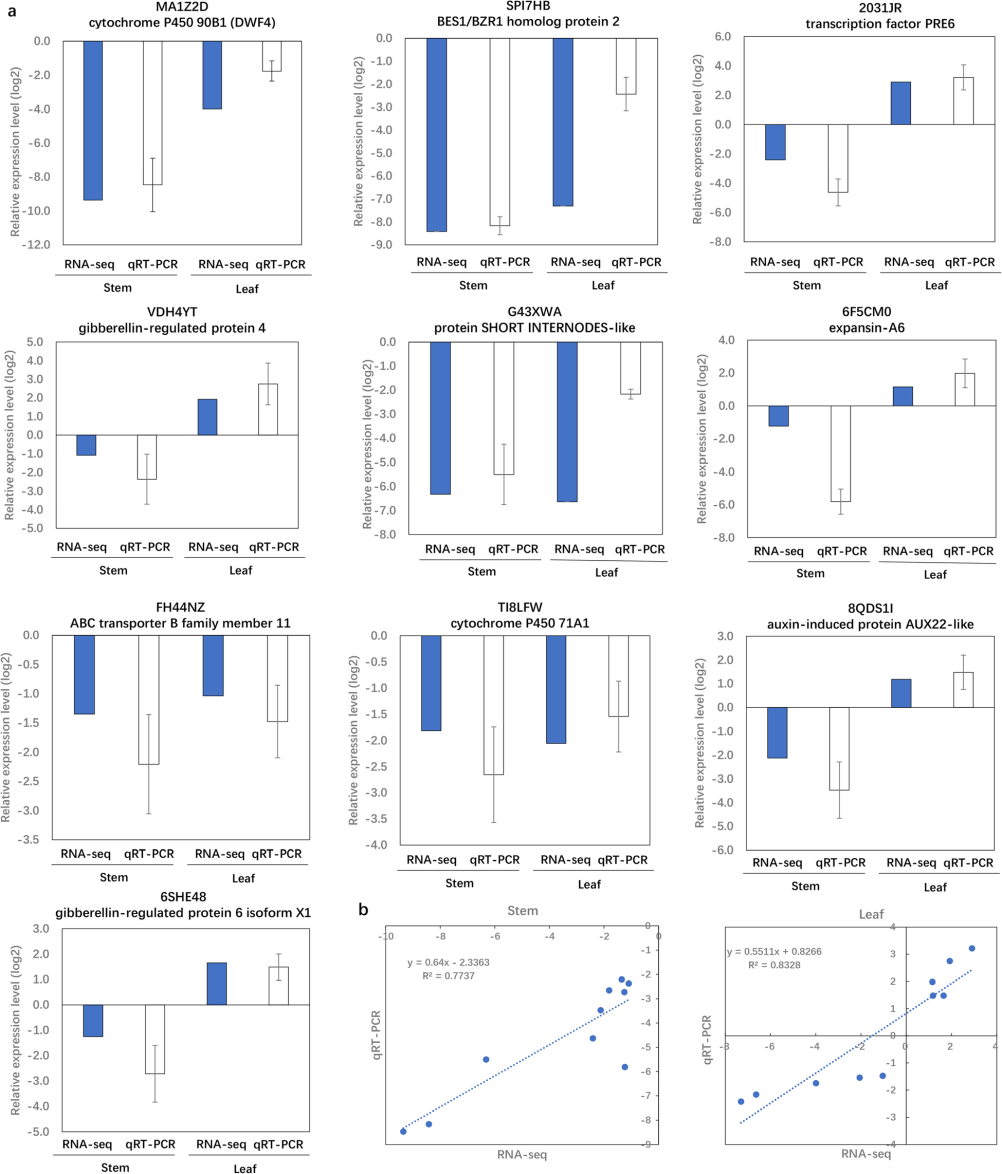
Verification of DEGs by qRT-PCR. **a**. Transcript levels of 10 genes related to hormone signal transduction and cell wall organization. Data are means of three replicates, and error bars represent±SE (*n* = 3). **b**. Pearson’s correlation of gene expression ratios between RNA-seq and qRT-PCR results. The correlation of the fold change was analyzed by RNA-seq (x-axis) with qRT-PCR (y-axis) data

In order to validate the RNA-Seq data, the expression levels of DEGs were detected using Quantitative Real-time PCR (qRT-PCR). A total of 10 genes related to hormone signal transduction and cell wall organization were selected (Fig. 6 and Additional file 9: Table S6). The relative expression levels (log_2_ *sdm2*/FH1) of these genes estimated by qRT-PCR were generally consistent with those by RNA-seq. The overall correlation coefficient of a liner regression analysis in stem and leaf was 0.7737 and 0.8328, respectively (Fig. 6).

## Discussion

Extensive studies indicated that deficiencies in cell wall-related genes, GA, BR and IAA signaling pathways all could result in dwarfish. These deficient mutants displayed dwarf or semi-dwarf phenotype with shorter hypocotyl or internode, smaller and dark-green leaves [4–7, 20–23, 40, 50, 69]. Microscopic assays indicated that the dwarf phenotypes of BR-deficient and BR-insensitive mutants were caused by reduced cell size [23, 32]. Maize *br2* mutant, deficient in the polar auxin transport, showed reduced stalk cell length and diameter [69]. In our study, *sdm2* phenotype was resulted from reduction both in internode number and internode length. The difference might be due to changes in cell division and cell expansion during the stem growth process, which was supported by the identification of a number of cell wall biosynthesis and metabolism enzyme encoding genes as DEGs. Cell division and cell expansion need the biogenesis or remodeling of cell wall. The extensibility of cell wall controls the rate of cell expansion. Genes, including *CesA* and *CSL*, responsible for cell-wall biogenesis, and *expansins* and *XTHs*, involved in cell wall loosening, are all critical in plant growth and development. Our transcriptome data revealed several *CesAs*, almost all *CSLs* and *expanin* genes were down-regulated in *sdm2* stem. Some *XTHs* were down-regulated in *sdm2* stem. Besides, genes encoding EGases which functions in the modification of hemicellulose network were down-regulated in *sdm2* stem. These results suggested that the cell wall biosynthesis and extensibility were reduced in *sdm2* stem, which might result in reduced cell division and cell expansion and further cause short internode and small number of stem nodes.

Studies revealed that many cell wall organization enzymes, including CesA, XTHs, EGases, and expansins were regulated by BRs [24, 45, 48, 70–72]. The essential roles of BRs in stem elongation have been confirmed by several mutants deficient in BR biosynthesis or perception [23, 30–32, 40]. Our results showed that key BR biosynthetic genes *CPD*, *DWF4* and *CYP90C1*/*D1* and *CYP71A1* were all down-regulated in *sdm2*. *CYP734A1*, which degrades BRs, was up-regulated in leaf of *sdm2.* The changing trends of above genes suggested the contents of BRs might be decreased in *sdm2*. BR response genes including *BRI1*, *BAK1* and *BES1*/*BZR1*, were down-regulated, especially *BES1*/*BZR1* in *sdm2* stem. These results suggested that BRs could be a key factor in the formation of semi-dwarf phenotype of *sdm2*. In addition, BR signaling pathway also regulate the leaf and seed size and shape. In Arabidopsis *cpd* mutant, the decrease of cell size and cell number resulted in reduced leaf size [73]. The seeds of Arabidopsis *det2* mutant were smaller and shorter because of decreased seed cavity, reduced endosperm volume and cell length and delayed embryo development [74]. Transgenic Arabidopsis and tobacco lines overexpressing *DWF4* displayed increased plant height, longer petiole and leaf blade length, increased number of branches and siliques compared to the control [75]. In this study, *sdm2* plants showed smaller leaf size, shorter petiole, and both decreased seed size and weight, which were all in accordance with phenotypic characteristics of BR-deficient mutants. We speculate that *CPD*, *DWF4*, *BRI1* and *BES1*/*BZR1* are key genes that lead to the phenotypic differences between *sdm2* and FH1 line.

In this study, several GA biosynthetic genes showed irregular change trends and some seemed to promote the biosynthesis of GAs, while all *GRP* genes decreased in *sdm2* stem which had the same expressional pattern with those of cell wall related genes. This result could be a reflection of lowered GA level in *sdm2* stem, and the up-regulated expression of GA20ox and 2-oxoglutarate-dependent dioxygenases might be resulted from the feedback regulation of GA signal pathway. The polar transport of auxin was essential for plant growth and development. In *sdm2*, the increased transcription of auxin influx carrier and down-regulation of auxin efflux carrier together with the irregular changes of auxin polar transporters, *MDR/ABCB* genes, suggested the disordered auxin transport from shoot apex and young leaves to stem. It might contribute to the formation of short internode in *sdm2*.

BRs could affect GA biosynthesis through positively regulating *GA20ox* expression [76]. DELLA protein could directly interact with BZR1 and inhibit its DNA binding ability, and the promotion of GAs on cell elongation require BZR1 or BRs [57]. Application of auxin could induce the expression of *GA20ox* and *GA3ox* while reduce the *GA2ox* transcript [77, 78]. Auxin and BRs also could interact with each other and synergistically regulate plant development [79, 80]. Auxin could directly induce *DWARF4* expression and BR biosynthesis [81]. In return, BRs influenced auxin redistribution through stimulating the expression of *PIN* genes [82]. There are complicated cross talk among hormones during plant growth and development. Our results suggested that the significantly down-regulated BR biosynthetic and response genes, low GA response and disordered auxin transport acted synergistically and contributed to the defects in *sdm2* stem growth.

**Fig. 7:**
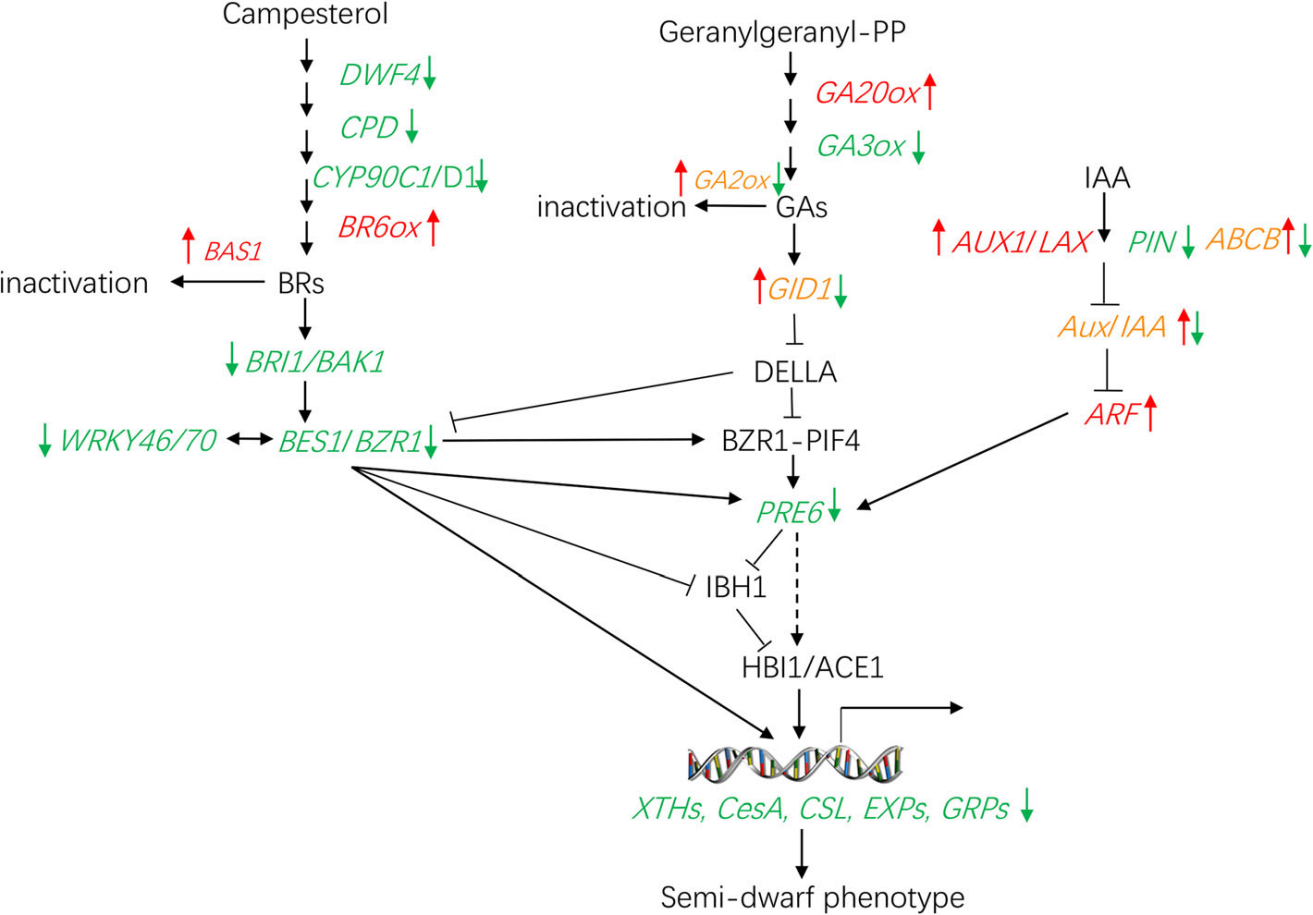
The regulatory network underlying semi-dwarf phenotype in *sdm2* mutant. DWF4: 22*a*-hydroxylase; *CPD*: putative C-3 oxidase; CYP90C1/D1: 3-epi-6-deoxocathasterone 23-monooxygenase; BR6ox: Brassinosteroid-6-oxidases; BAS1: Brassinosteroids C-26 hydroxylase; *BRI1*: Brassinosteroid insensitive 1; BAK1:BRI1-associated receptor kinase 1; BES1: *BRI1*-EMS suppressor 1; BZR1: Brassinazole resistant 1; GA20ox: GA 20-oxidases; GA3ox: GA 3-oxidases; GA2ox: GA 2-oxidases; GID1: GA-insensitive dwarf 1; DELLA: proteins containing conserved DELLA domains in the N terminus; PRE6: Paclobutrazol resistant 6; IBH1: ILI1 binding bHLH protein 1; HBI1: homolog of BEE2 interacting with IBH1; ACE1: activator for cell elongation 1; PHY: phytochrome; PIFs: phytochrome-interacting factors; AUX1/LAX: auxin1/like-AUX1; PIN:*PIN-formed protein; ABCB:* subfamily B of ATP-binding cassette; Aux/IAA: auxin resistant/indole-3-acetic acid inducible; ARF: auxin response factor; CesA: cellulose synthases; CSL: cellulose synthase-like genes; XTHs: xyloglucanendotransglucosylase/endohydrolases; EXPs: expansins; GRPs: gibberellin-regulated proteins

As the common target genes of the core transcription module, PRE family factors connect external and endogenous signals with downstream cell elongation components together through an antagonistic cascade reaction. The expressions of *PRE6* genes in *sdm2* stem and leaf were consistent with those of cell wall enzymes, which further proved the direct promotion effects of PRE on cell elongation. Tracing back to the upstream hormonal signals, the extremely low expression of BR biosynthetic and signal transduction genes and low GA signaling in *sdm2* stem all contributed to the down-regulation of *PRE* genes, and subsequently reduced cell elongation factors which finally caused the semi-dwarf phenotype. Based on these results, we proposed a potential model regulating peanut stem development (Fig. 7). BRs, GAs and auxin signaling pathways interact cooperatively to influence *PRE* expression, and regulate downstream cell wall-related genes to control cell elongation during stem development. However, further research is required to verify this hypothesis after cloning the mutant gene.

## Conclusions

The transcriptome analysis of *sdm2* identified a number of differentially expressed genes involved in hormone biosynthesis, signaling and cell wall synthetic and metabolic pathways. Especially several genes in BR pathway were significantly down-regulated in stem and leaf of *sdm2* as compared to FH1. Genes in cell wall synthetic and metabolic pathway which related to cell elongation were generally down-regulated in *sdm2* stem. These findings provide critical information for uncovering the molecular genetic control of peanut stem development.

## Methods

### Plant materials

The cultivated peanut cultivar Fenghua1 (FH1) was bred by and acquired from Yongshan Wan’s laboratory in Shandong Agricultural University, Shandong Province of China. In 2013, the seeds of FH1 was irradiated with ^60^Co γ-ray of 500 Gy. In M_2_ generation, a line with phenotypic segregation of normal and semi-dwarf plant height was screened. After self-crossing for another two generations, the stable semi-dwarf peanut line was obtained and named *semi-dwarf mutant 2* (*sdm2*).

### Planting conditions and sampling methods

In 2018, *sdm2* and its wild type FH1 were sowed in experimental farm of Shandong Academy of Agricultural Sciences. The ridging mode was adapted with ridge spacing of 90 cm, ridge width of 55 cm and ridge height of 12 cm. Peanut seeds were sowed for two rows on each ridge with a single seed per hole, and the plant spacing and row spacing were 18 cm and 35 cm, respectively. All field management followed the standard agricultural practices. For each line, about 100 plants were planted. It was sowed on May 1st and harvested on September 5th with the growing period of 129 days. The days after planting (DAP) was used to calculate seedling growth time. During the vegetative period, at least 5 plants from each line were selected to investigate the main stem height through measuring the length from base of the above-ground plant to the tip of the main stem for every 2 weeks. The 1~5 internodes and the first fully developed leaf were collected from 60 DAP plants of *sdm2* and FH1 for RNA sequencing. At this time, the main stem height of *sdm2* and FH1 was significantly different. The samples were frozen immediately in liquid nitrogen, and stored at − 80 °C for RNA extraction. For both stem and leaf, three replicates were prepared.

The hydroponic culture experiment was conducted in illumination incubator in the laboratory. The temperature was 32 °C and the light-dark cycle was 14 h of light and 10 h of darkness. Seeds of *sdm2* and FH1 were planted in glass ware filled with Hogland culture solution with the preservative film for support. About 20 plants were planted for each line. At the 14 DAP, the number of internodes and the length of each internode and hypocotyl were measured. The main stem height was the summation of each internode length. Each line had five replicates.

### RNA extraction, cDNA synthesis and high throughput sequencing

Total RNA was extracted from stem and leaf using Trizol Reagent (TaKaRa, Inc., Dalian, China) according to the manufacturer’s instructions. RNA samples were treated with DNase I to remove genomic DNA contamination. RNA quality and quantity were analyzed using Agilent 2100 and NanoDrop. mRNA was enriched using magnetic beads with Oligo (dT) and cleaved into short fragments (~ 200 nt) in fragmentation buffer. The reverse transcription was conducted with random hexamer primer and then the second strand cDNA was synthesized. After end repair, the 5′ tails were phosphorylated, the 3′ tails were added with anadenine. Sequencing adaptors were ligated to the double-stranded DNA fragments. Then the fragments were amplified by PCR to construct cDNA library. The library was sequenced using BGISEQ-500 platform by Beijing Genomics Institute (BGI).

### Bioinformatics analysis of RNA-Seq data

Raw reads were generated from each cDNA library. To obtain clean reads, the adaptor sequences, low-quality reads and reads containing more than 5% unknown bases were removed using SOAPunke and trimmomatic software. All clean reads were mapped to the reference genome of *Arachis hypogaea* cv. Tifrunner (https://www.peanutbase.org/data/public/Arachis_hypogaea/) using HISAT2 program [83]. The clean reads were aligned to reference gene by Bowtie2 (RNA-Seq by Expectation Maximization) [84]. The statistical analyses of randomness, degree of coverage, and sequencing saturation were also accomplished. The gene expression level was calculated with RSEM method [85] and normalized to FPKM (Fragments Per Kb per Million reads). The relative gene expression level between two samples was counted by log_2_ ratio. Pearson’s correlation coefficient between every two samples and principal component analysis (PCA) were performed. Differentially expressed genes (DEGs) were identified using DEGseq2 method and screened with the criteria of fold change≥2 and Q-value≤0.001 [86].

Gene Ontology (GO) annotation was carried out by Blast2GO program through comparing DEGs with GO terms in the GO database and GO functional classification was performed using WEGO software. KEGG (Kyoto Encyclopedia of Genes and Genomes) pathway analysis was conducted by mapping DEGs to KEGG database. The GO and KEGG functional enrichment were performed using hypergeometric test. The *p*-value formula in hypergeometric test can be acquired from https://en.wikipedia.org/wiki/Hypergeometric_distribution in detail. FDR (False Discovery Rate) correction of all *p*-values was conducted. The GO terms and KEGG pathways whose FDR ≤ 0.01 were defined as significant enriched. BLASTX (E < 0.00001) against NCBI Nr database was carried out. For transcription factor (TF) annotation, the ORF of DEGs were detected by getorf software, then these ORFs were aligned to TF protein domain (data from PlantTFDB) using hmmsearch program, and finally TFs were identified according to the TF family characteristics described in PlantTFDB (http://plntfdb.bio.uni-potsdam.de/v3.0/). The DEGs were also aligned to Plant Resistance Gene Database (PRGdb, http://prgdb.crg.eu/) by DIAMOND software to identified the resistance genes according to the query coverage and identity. The heatmap of cell wall related gene expression was charted using MeV software (https://sourceforge.net/projects/mev-tm4/) with the data of relative gene expression level between two samples counted by log_2_ (*sdm2*/FH1).

### qRT-PCR validation of RNA-Seq data

We used qRT-PCR method to verify the expressional levels of 10 selected genes. RNA samples were those used for high-throughput sequencing and the reverse transcriptions were performed using PrimeScript II 1st Strand cDNA Synthesis Kit (TaKaRa). The gene-specific primers were designed using PerlPrimer software and were listed in Additional file 10: Table S7. We performed qRT-PCR reaction on ABI7500 Real Time System (Applied Biosystems) using TB Green™ Premix Ex Taq™ II (TaKaRa). The parameters of thermal cycle were 94 °C for 10 min, followed by 40 cycles of 94 °C for 15 s and 60 °C for 1 min in a 20 μl volume. Each reaction was performed three biological replications with *actin* gene as internal reference gene. The relative expressional level of each gene between *sdm2* and FH1 was calculated by 2^-△△Ct^ method.

## Supplementary information

**Additional file 1 Figure S1.** The correlation heatmap of each sample of *sdm2* and FH1. The X and Y axes represent each sample. The color represents the Pearson correlation coefficient (the darker the color, the higher the correlation; the lighter the color, the lower the correlation).

**Additional file 2 Figure S2.** The principal component analysis of each sample of *sdm2* and FH1. X axis represents the principal component 1 and Y axis represents the principal component 2.

**Additional file 3 Table S1.** Differentially expressed genes of stem between *sdm2* and FH1.

**Additional file 4 Table S2.** Differentially expressed genes of leaf between *sdm2* and FH1.

**Additional file 5 Table S3.** GO classification of DEGs in stem and leaf.

**Additional file 6 Figure S3.** Bubble diagram of top 20 enriched GO terms of DEGs in stem and leaf. X axis represents the Rich Ratio, which meaning the ratio of selected gene number annotated to a particular item to the total number of genes in this item in one species. The calculating formula is Rich Ratio = Term Candidate Gene Num/Term Gene Num. Y axis represents GO Term. The size of the bubbles indicates the number of genes annotated to a GO Term. And the color represents Q-value of enrichment. The deeper the color, the smaller the Q-value.

**Additional file 7 Table S4.** The top 20 enriched GO terms of DEGs in stem and leaf.

**Additional file 8 Table S5.** The top 20 enriched KEGG pathways of DEGs in stem and leaf.

**Additional file 9 Table S6.** Validation of RNA-seq results via qRT-PCR.

**Additional file 10 Table S7.** Primers for qRT-PCR analysis.

## Data Availability

The RNA-seq data in this study were available at NCBI Short Read Archive with the accession number of SRP213768 (https://www.ncbi.nlm.nih.gov/search/all/?term=SRP213768).

## Abbreviations

ABCB: Subfamily B of ATP-binding cassette
ACE1: Activator for cell elongation 1
ARF: Auxin response factor
Aux/IAA: Auxin resistant/indole-3-acetic acid inducible
AUX1/LAX: Auxin1/like-AUX1
BAK1: BRI1-associated receptor kinase 1
BAS1: Brassinosteroids C-26 hydroxylase
BES1: BRI1-EMS suppressor 1
BR6ox: Brassinosteroid-6-oxidases
BRI1: Brassinosteroid insensitive 1
BZR1: Brassinazole resistant 1
CesA: Cellulose synthases
CPD: Putative C-3 oxidase
CSL: Cellulose synthase-like genes
CYP90C1/D1: 3-epi-6-deoxocathasterone 23-monooxygenase
DELLA: Proteins containing conserved DELLA domains in the N terminus
DWF4: 22a-hydroxylase
EXPs: Expansins
GA20ox: GA 20-oxidases
GA2ox: GA 2-oxidases
GA3ox: GA 3-oxidases
GID1: GA-insensitive dwarf 1
GRPs: Gibberellin-regulated proteins
HBI1: Homolog of BEE2 interacting with IBH1
IBH1: ILI1 binding bHLH protein 1
PCA: Principal component analysis
PHY: Phytochrome
PIFs: Phytochrome-interacting factors
PIN: PIN-formed protein
PRE6: Paclobutrazol resistant 6
qRT-PCR: Quantitative Real-time PCR
SAUR: Small auxin up RNA
XTHs: Xyloglucanendotransglucosylase/endohydrolases
