# Supplementary figures and images for: Transcriptome profiling provides insights into molecular mechanism in Peanut semi-dwarf mutant
Source: BMC Genomics. 2020 Mar 5;21:211. doi: 10.1186/s12864-020-6614-0 (PMC7059693; doi:10.1186/s12864-020-6614-0)

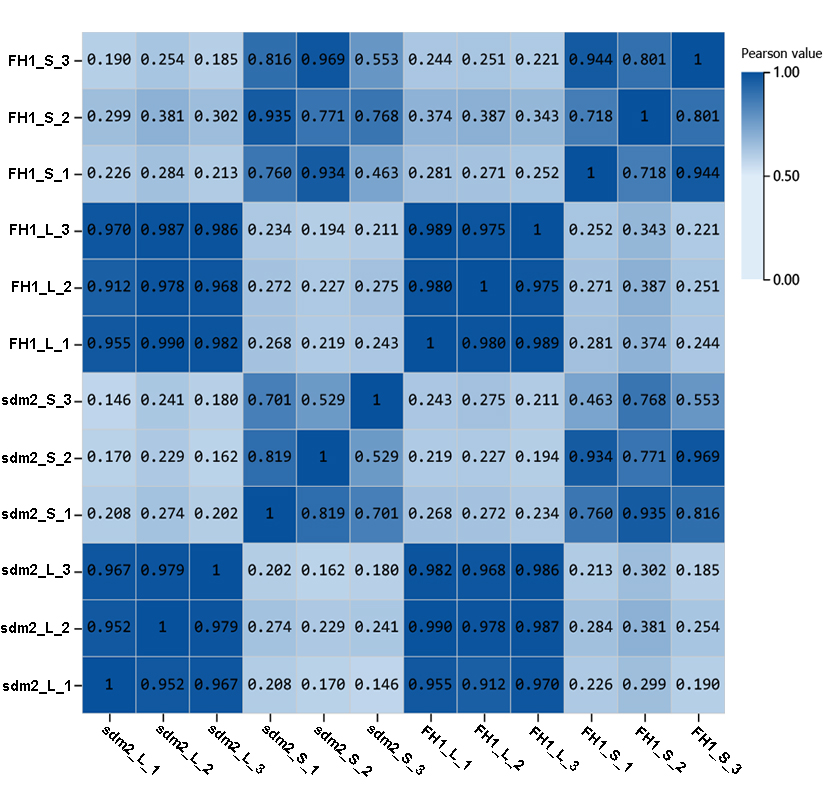

Supplement: Supplementary file 1 — Additional file 1 Figure S1. The correlation heatmap of each sample of sdm2 and FH1. The X and Y axes represent each sample. The color represents the Pearson correlation coefficient (the darker the color, the higher the correlation; the lighter the color, the lower the correlation). [file 12864_2020_6614_MOESM1_ESM.jpg]

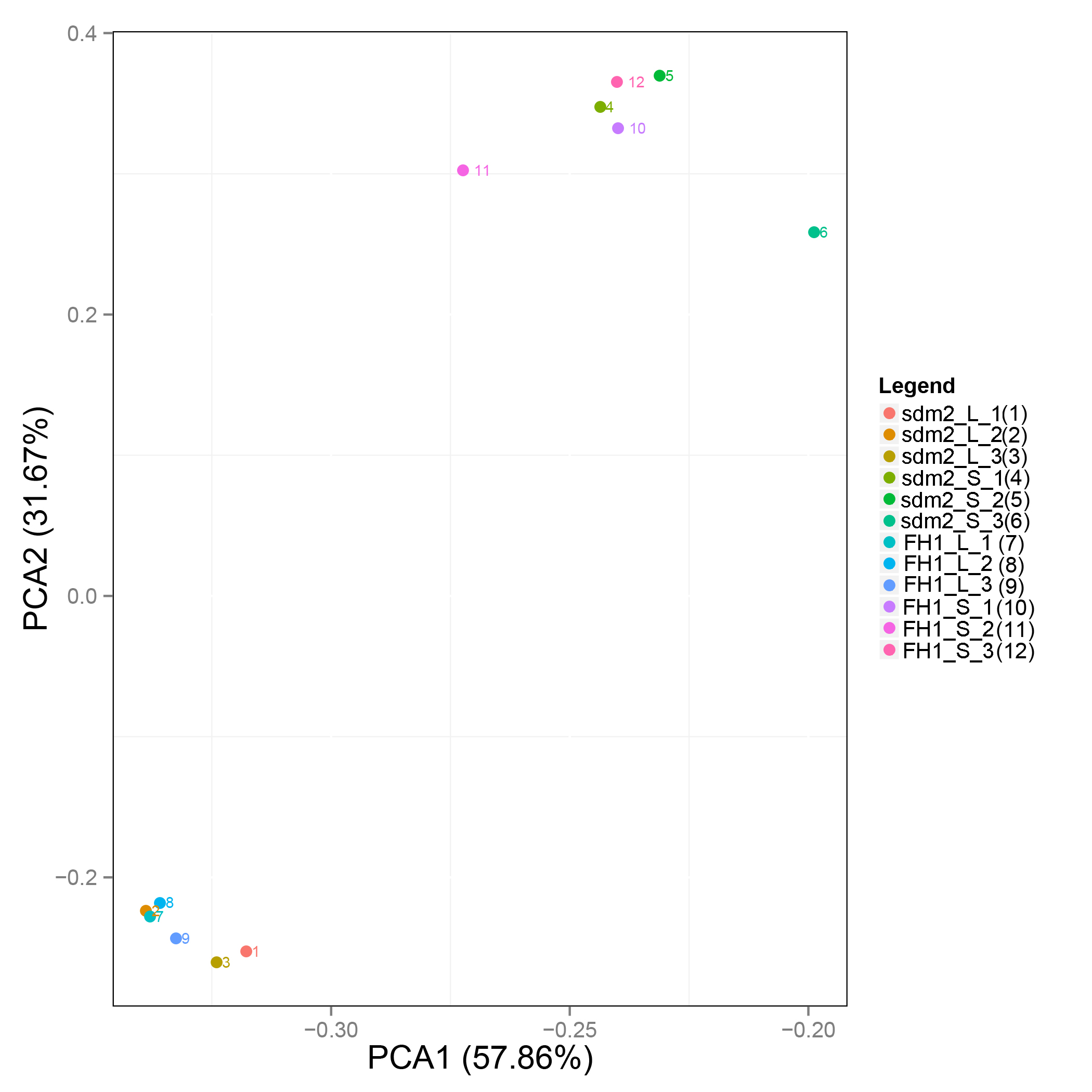

Supplement: Supplementary file 2 — Additional file 2 Figure S2. The principal component analysis of each sample of sdm2 and FH1. X axis represents the principal component 1 and Y axis represents the principal component 2. [file 12864_2020_6614_MOESM2_ESM.jpg]

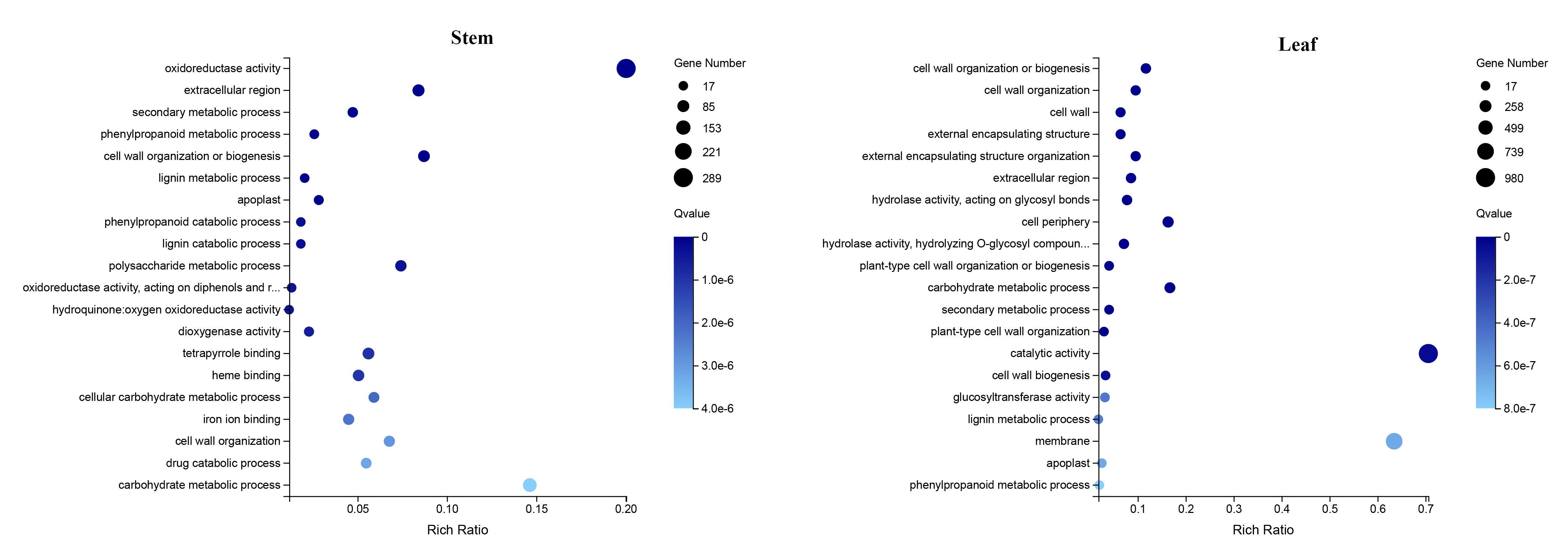

Supplement: Supplementary file 6 — Additional file 6 Figure S3. Bubble diagram of top 20 enriched GO terms of DEGs in stem and leaf. X axis represents the Rich Ratio, which meaning the ratio of selected gene number annotated to a particular item to the total number of genes in this item in one species. The calculating formula is Rich Ratio = Term Candidate Gene Num/Term Gene Num. Y axis represents GO Term. The size of the bubbles indicates the number of genes annotated to a GO Term. And the color represents Q-value of enrichment. The deeper the color, the smaller the Q-value. [file 12864_2020_6614_MOESM6_ESM.jpg]
